# Differential Effects of *Bartonella henselae* on Human and Feline Macro- and Micro-Vascular Endothelial Cells

**DOI:** 10.1371/journal.pone.0020204

**Published:** 2011-05-27

**Authors:** Moez Berrich, Claudine Kieda, Catherine Grillon, Martine Monteil, Nathalie Lamerant, Julie Gavard, Henri Jean Boulouis, Nadia Haddad

**Affiliations:** 1 Unité Mixte de Recherche de Biologie Moléculaire et Immunologie Parasitaires et Fongiques, Ecole Nationale Vétérinaire d'Alfort, Université Paris-Est, Maisons-Alfort, France; 2 Unité Propre de Recherche 4301, Centre de Biophysique Moléculaire, Centre National de la Recherche Scientifique, Orléans, France; 3 Unité 1016 Inserm, Institut Cochin, CNRS, Université Paris Descartes, Paris, France; Max Planck Institute for Infection Biology, Germany

## Abstract

*Bartonella henselae*, a zoonotic agent, induces tumors of endothelial cells (ECs), namely bacillary angiomatosis and peliosis in immunosuppressed humans but not in cats. *In vitro* studies on ECs represent to date the only way to explore the interactions between *Bartonella henselae* and vascular endothelium. However, no comparative study of the interactions between *Bartonella henselae* and human (incidental host) ECs *vs* feline (reservoir host) ECs has been carried out because of the absence of any available feline endothelial cell lines.

To this purpose, we have developed nine feline EC lines which allowed comparing the effects of *Bartonella* strains on human and feline micro-vascular ECs representative of the infection development sites such as skin, *versus* macro-vascular ECs, such as umbilical vein.

Our model revealed intrinsic differences between human (Human Skin Microvascular ECs –HSkMEC and Human Umbilical Vein ECs – iHUVEC) and feline ECs susceptibility to *Bartonella henselae* infection.

While no effect was observed on the feline ECs upon *Bartonella henselae* infection, the human ones displayed accelerated angiogenesis and wound healing.

Noticeable differences were demonstrated between human micro- and macro-vasculature derived ECs both in terms of pseudo-tube formation and healing. Interestingly, *Bartonella henselae* effects on human ECs were also elicited by soluble factors.

Neither *Bartonella henselae*-infected Human Skin Microvascular ECs clinically involved in bacillary angiomatosis, nor feline ECs increased cAMP production, as opposed to HUVEC.

*Bartonella henselae* could stimulate the activation of Vascular Endothelial Growth Factor Receptor-2 (VEGFR-2) in homologous cellular systems and trigger VEGF production by HSkMECs only, but not iHUVEC or any feline ECs tested.

These results may explain the decreased pathogenic potential of *Bartonella henselae* infection for cats as compared to humans and strongly suggest that an autocrine secretion of VEGF by human skin endothelial cells might induce their growth and ultimately lead to bacillary angiomatosis formation.

## Introduction

Since its discovery in 1992 [1], *Bartonella henselae* (*B. henselae*) is considered as the main agent of cat scratch disease (CSD) in humans and is also responsible for a growing number of diseases in patients [2]. This facultative intracellular bacterium is now known to be responsible for more serious diseases in both immunocompetent patients,*e.g.* endocarditis, and immunosuppressed patients, such as bacillary angiomatosis and peliosis [3], characterized by pseudotumoral proliferation of endothelial cells (ECs). These unusual vascular lesions occur mainly or exclusively in the skin, liver and spleen [3]. Cats are the main reservoir of this zoonotic bacterium [4]. However, as compared to humans, normal or immunosuppressed cats display high rates of sub-clinical infections and remain usually healthy, with only chronic bacteraemia [4], [5], [6]. In addition, in cats, *B. henselae* infection has not yet been associated with bacillary angiomatosis or peliosis [7], [8].

Two genotypes (I and II) of *B. henselae* have been described on the basis of 16S rRNA sequence analysis [9]. Epidemiological studies strongly suggest that genotype I is more virulent in humans than genotype II [9], [10], [11], [12], [13]. In particular, only genotype I has been found associated to date to bacillary angiomatosis and peliosis [14], but this observation has to be confirmed by further studies.

The presence of *B. henselae* micro-colonies adjacent to proliferating endothelial cells has been histologically demonstrated, and suggested that *Bartonella*/ECs interactions might trigger a proangiogenic process, potentially leading to vascular lesions [15]. Due to the lack of any appropriate animal model, cultured ECs provide tools to study the interactions between *B. henselae* and the vascular endothelium.

These approaches have been useful for identifying *B. henselae* virulence factors. *B. henselae* adhesin A (BadA) (originally described as “pilus”) [16] is important for pathogenicity [17], being involved in the adhesion to extracellular-matrix proteins and to ECs. It activates hypoxia-inducible factor-1 and pro-angiogenic cytokines secretion [18]. Recently, the VirB/VirD4 type IV secretion system and subsets of its translocated *B. henselae* effector proteins (BepA and BepG) were found to modulate the angiogenic activity of *B. henselae* [19], [20]. Other studies have suggested that the process through which *B. henselae* triggers ECs proliferation involved released or secreted bacterial factors [21], [22], [23], [24].

Host factors have also been found *in vitro* to play a role in *B. henselae* driven angiogenesis. VEGF (Vascular Endothelial Growth Factor) is known as the main angiogenic factor, which positively regulates migration, proliferation and survival of endothelial cells and has been shown to be over-secreted in the tumor micro-environment [25]. According to McCord et *al* [26], ECs infected by *B. henselae* Houston I may upregulate expression and production of pro-angiogenic proteins. Studies of VEGF expression in clinical samples [27] or *in vitro* [22], [27], [28], suggest a paracrine loop type of VEGF activity. Moreover, the anti-apoptotic activity of *B. henselae* BepA, in human umbilical vein endothelial cells (HUVEC), correlates with an important elevation of intracellular adenosine 3′, 5′-cyclic monophosphate (cAMP) level [29]. A more recent study demonstrated that *B. henselae* infection involves the intrinsic apoptotic pathway [30].

ECs are morphologically and functionally heterogeneous with major differences between those from the macro- *versus* micro-circulation as documented for a variety of tissues [31], [32], [33]. Except rare cases where ECs of the microvasculature have been included in *Bartonella* infection *in vitro* experiments [26], [28], [30], [34], studies are mostly based on the use of primary HUVEC or other macrovasculature-derived cells like the hybrid EA.Hy.926. These cells originate from a large vessel of the placenta and are very different from microvasculature-derived ECs [31], [32], [33], [35] clinically involved in bacillary angiomatosis and peliosis.

In addition, primary ECs will not allow providing repeatable and reproducible data, as these cultures lead to highly activated cells, in limited amounts and for a short term. Cell lines, established in a controlled identical manner, represent the best alternative to overcome these problems.

No comparative studies on the interactions between *B. henselae* and human (incidental host) ECs *versus* feline (reservoir host) ECs have ever been undertaken, because of the absence of any available feline ECs. Hence, feline ECs lines were developed and the *in vitro* effects of infection by distinct *Bartonella* species*/*strains (*B. henselae* genotype I/II and *B. tribocorum*) on human *versus* feline ECs derived from the macro- and micro-vasculature were compared. By studying various parameters as *in vitro* angiogenesis, stimulation of wound-healing, induction of cAMP, production of VEGF and activation of VEGF Receptor-2 (VEGFR-2), we demonstrated the crucial significance of the tissue origin of ECs and the specificity of the relationship between various strains of *B. henselae* with human *versus* feline cell lines. Our work contributes to the understanding of the differential outcomes of *B. henselae* infection in humans *versus* cats.

## Materials and Methods

### Human endothelial cell lines

Two immortalized lines of human organospecific ECs previously developed [31] were used in this study: one isolated from macrovasculature (immortalized Human Umbilical Vein Endothelial Cells: iHUVEC) and one isolated from the skin microvasculature (Human Skin Microvascular Endothelial Cells: HSkMEC) [36].

### Feline endothelial cell lines

#### Establishment

Four immature and not viable embryos were aseptically removed at the School of Veterinary Medicine, Alfort, France, in the reproductive diseases service by therapeutic caesarean from queen after 45 days of gestation, and organs or tissues biopsies were taken from each embryo. Tissues and organs were placed in RPMI medium (Gibco, BRL, Cergy Pontoise France) without fetal bovine serum (FBS), supplemented by antibiotics and stored at 4°C.

Isolation of macrovascular (from umbilical vein FOmEC) or microvascular (from Skin FSkMEC, Peripheral Lymph Node FPLNMEC, Lung FLuMEC, Brain FBrMEC, Liver FLiMEC, Peyer's patches FPPMEC, Intestine FIntMEC, Heart FHeMEC) ECs was performed [31], [37], [38]. Immortalization was realized as already described for human endothelial cells [31]. Lines were characterized, patented (CNRS-ENVA, French patent N° 10/00094) and deposited at the Collection Nationale de Cultures de Microorganismes (Institut Pasteur, Paris, France).

The feline ECs lines were cultured in OptiMEM medium (Gibco BRL,Cergy Pontoise, France) +2% of FBS+0.2% of fungizone+0.4% of gentamicin (Gibco BRL) and were maintained in a humidified atmosphere containing 5% carbon dioxide (CO_2_) at 37°C.

### Characterization

#### a- Intracellular von Willebrand factor (vWF) and Angiotensin-Converting Enzyme (ACE) detection

Established cell lines were grown on twelve-well microscope slides (ICN Bio-medicals, Aurora, OH, USA) for 48 h. Monolayers were fixed for 10 min with paraformaldehyde (PFA, 2%) and permeabilized, for 30 min at 37°C, with Triton X100 (0,05%) (Sigma, France).

For intracellular vWF detection, a rabbit polyclonal anti-human vWF antibody (A0082-Dako) (20 µg/ml), was applied to the cells for 2 h at RT and washed twice with c-PBS containing 1% bovine serum albumin (c-PBS-BSA). The second antibody was a goat Fluorescein IsoThioCyanate (FITC)-anti-rabbit immunoglobulin antibody (20 µg/ml) (SBE, CliniSciences, Montrouge, France), and cells were further incubated for overnight at 4°C.

For ACE detection, polyclonal rabbit anti-human ACE antibody (sc 20791- Santa Cruz Biotechnology) and, further, a secondary goat Fluorescein IsoThioCyanate (FITC)-anti-rabbit immunoglobulin antibody (SBE, CliniSciences, Montrouge, France) was used. Cells were examined by fluorescence microscopy (Leica DMI 4000 B).

#### b- Angiogenesis assays

A 96-well plate (Falcon, BD Biosciences, Grenoble, France) was coated with Matrigel™ (BD Biosciences) mimicking the extracellular matrix (40 µl by well). The Matrigel™ was allowed to polymerize for 1 h at 37°C before cell seeding (8×10^3^ cells/well). ECs rearrangements and capillary-like structure formation were observed each hour until the 24^th^ hour and then every day until the 7^th^ day. It was photographed regularly under a microscope (Nikon TMS, Japan)

Monkey kidney epithelial cells (Vero line, ATCC, CCL-81) were used as a non endothelial cell control.

### Bacterial strains

Four strains of *B. hensela*e were used, based on their species origin (human or feline) and genotype (I or II). The two strains isolated from human patients were the reference strain Houston-1 (genotype I (H1) / ATCC 49882) [1] and the genotype II Marseille strain (H2) kindly supplied by Jean Marc Rolain (Unité des rickettsies, Marseille, France). The two strains isolated from cats were a genotype I strain (F1/ Strain 297172) kindly supplied by Bruno Chomel (University of California, Davis, USA) and a genotype II *B. henselae* strain initially isolated in our laboratory (F2). *B. tribocorum* (Bt) (CIP 105476 T), isolated from rat and without known pathogenicity for human and cats was also used.

All *Bartonella* strains were cultured on sheep blood agar medium (BioMerieux, Craponne, France) for 5 to 7 days in humidified atmosphere at 35°C and 5% CO_2_.

To investigate the specificity of *Bartonella*-triggered angiogenesis, we used *Escherichia coli* (*DH5α*) (Invitrogen, France) as a negative control. This bacterium was grown at 37°C on Luria-Bertani (LB) agar or in LB broth (Sigma, France). Heat-inactivated *B. henselae* were used in order to check if dead bacteria were still able to stimulate ECs angiogenesis. The inactivation of heat-treated bacteria (56°C, 30 min) was checked on sheep blood agar medium.

### Generation of *Bartonella* culture supernatants

*B. henselae* strains and *B. tribocorum* were harvested from blood agar plates and suspended in Schneider medium 1x (Gibco, France). The bacterial cultures were gently shaken in Schneider medium for 18 h at 200 rpm and 37°C on an orbital shaker (Innova 4230, New Brunswick Scientific, Edison, NJ, USA). After 18 h of incubation, the suspensions were removed from the flasks and spun at 1000 g and 4°C for 10 minutes to form a soft pellet. The supernatants were removed and passed through a 0.22 µm filter to remove all bacteria. The bacteria-free supernatants were dialyzed with OptiMEM and concentrated in a Centrifugal filter (Amicon ® Ultra-15 Millipore, France). Culture supernatant control medium was also generated through similar methods. Protein concentrations were determined by bicinchoninic acid assay (BC Assay Kit, Uptima, Interchim, France).

### *In vitro* induction of angiogenesis

Twenty-four hours before infection of ECs (iHUVEC -immortalized Human Umbilical Vein Endothelial Cells-, HSkMEC -Human Skin Microvascular Endothelial Cells-, FOmEC-Feline Umbilical vein Macrovascular Endothelial Cells and FSkMEC-Feline Skin Microvascular Endothelial Cells), antibiotics were removed from the culture medium. ECs (8×10^3^ cells/well) were infected with *Bartonella* at a multiplicity of infection (M.O.I.) of 50, 100 and 150 bacteria per cell or treated with bacteria culture supernatant (protein concentration: 250 µg/ml) and then were seeded on 96 well plates previously coated with Matrigel™ (BD Biosciences, Grenoble, France). Videos for ECs rearrangement and capillary-like structure formation were acquired and registered for image analysis (Zeiss axiovision program). The Zeiss axiovert 200 M, videomicroscope equipped for temperature, gas and humidity controls was used to acquire images in time lapse conditions and reconstitute the kinetics of the angiogenesis dynamic process. The pseudo-vessel formation was monitored continuously during the first 24 hours and then observed every day during the following 7 days.

Capillary-like structure formation was quantified by measuring the length of tubes and counting the branching points [39].

### Healing test

iHUVEC, HSkMEC, FOmEC and FSkMEC were seeded in 24-well plates at a density of 10^5^ cells/well and allowed to attach overnight in OptiMEM medium without antibiotics. When cells came to confluence, a wound was made in the center of the well using the extremity of a sterile pipette tip. Then, ECs were infected with bacteria (100 M.O.I.) for 24 h. The healing was observed and photographed under a Nikon TMS microscope. Healing distances (in µm) were measured at time 0 and 24 h after the wound.

### Determination of intracellular cAMP level

After infection of ECs (iHUVEC, HSkMEC, FOmEC and FSkMEC) by *Bartonella* strains with 150 M.O.I.for 30 h in 24 well plates, cells were washed with PBS and lysed. Intracellular cAMP levels were determined by the EIA system (Biotrak, Amersham, Biosciences, France) as described by the manufacturer.

### Measurement of VEGF levels in conditioned medium (ELISA)

VEGF concentration in culture medium was measured using a commercially available human VEGF ELISA Kit (R&D Systems, Minneapolis, MN, USA) according to the manufacturer's instructions. Briefly, cells were plated at a density of 5×10^4^ cells/0,5 ml/well in a 24 well plate and 12 h later cells were infected with *Bartonella* at a M.O.I. of 100. Conditioned medium was collected 72 h after infection, cellular debris and extracellular bacteria removed by centrifugation, and the medium was kept at −80°C until VEGF quantitation was undertaken. Cell number was determined immediately after medium recovery.

### Western blot analysis of VEGF-R2

24 h after infection (M.O.I.of 100), iHUVEC, HSkMEC, FOmEC and FSkMEC were lysed in TNT lysis buffer (10 mM Tris-HCL pH 7.5; 150 mM NaCL; 1% Nonidet P-40; 1% Triton X100 and 2 mM EDTA) supplemented with protease and phosphatase inhibitors (10 µg/ml leupeptin and apropotin; 1 mM phenylmethylsulfonyl fluoride; 25 mM NaF and 300 µM vanadate) for 15 min at 4°C. Equal amounts of samples were subjected to SDS-polyacrylamide gel electrophoresis and transferred onto a polyvinylidene difluoride membrane. Membranes were incubated with either anti-VEGFR-2 or anti-phosphorylated Y1175 VEGFR-2 (Cell signaling, Ozyme, Saint-Quentin-en-Yvelines, France). Daylight680 fluorescent dye-conjugated secondary antibody was used for revelation and membranes were scanned using the Odyssey Infra-Red Imaging System (Li-Cor BioSciences, EuroSep, Cergy-Pontoise, France).

### Statistical analysis

All experiments were performed at least three times (except VEGF production: twice) and gave comparable results. Differences between mean values of experimental and control groups were analyzed by Student's t-test. *P*-values less than 0.05 were considered to be statistically significant. The data were expressed as mean ± standard deviation (SD).

## Results

### Establishment of new feline endothelial cell lines

In order to compare the feline ECs with their human counterparts (i.e HSkMEC – Human Skin Microvascular EC - and iHUVEC – immortalized Human Umbilical Vein EC -), two feline cell lines (FSkMEC - Feline Skin Microvascular EC - and FOmEC- Feline Umbilical Macrovascular EC -) were selected among the nine established feline ECs lines which have been characterized for EC markers as vWf and ACE (data not shown).

**Figure 1:**
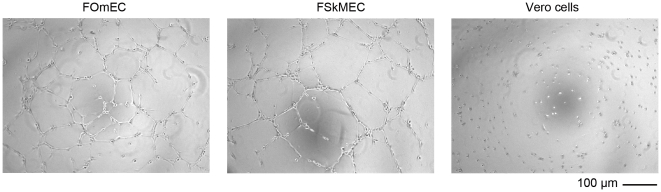
*In vitro* angiogenesis of feline ECs 10 h after seeding on Matrigel™. Feline ECs are able to form pseudovessels. FOmEC: Feline Umbilical Vein Endothelial Cells; FSkMEC: Feline Skin Microvascular Endothelial Cells. Vero cells: Monkey kidney epithelial cells used as non-endothelial control cells. Original magnification: 50x.

Moreover, because ECs can retain *in vitro* their ability to undergo angiogenesis, feline ECs lines were tested for their potentiality to form capillary-like structures in the classical Matrigel™ assay. All feline ECs were capable of performing angiogenesis on Matrigel™. Figure 1 shows angiogenesis observed with FSkMEC and FOmEC.

### Angiogenic response of ECs to *Bartonella* infection

Capillary-like structures of uninfected feline ECs (Fig. 1) began to form within two hours after seeding on Matrigel™ as compared to uninfected human ECs, while occurring within approximately five hours for macrovasculature derived ECs (iHUVEC) and within 10 hours for microvasculature ECs (HSkMEC) (table 1). The network of pseudo-vessels persisted in human ECs, until 24 hours for macro-vasculature derived ECs and until 7 days for microvascular ECs. Of note this network disappeared only after 20 hours with feline ECs (table 1).

**Table 1:** Effect of *Bartonella* on ECs angiogenesis kinetics.

		Feline EC		Human EC	
		unifected	infected	uninfected	infected
Beginning of angiogenesis	Macrovascular	2 h	2 h	5 h	5 h
	Microvascular	2 h	2 h	10 h	8 h
End of angiogenesis	Macrovascular	20 h	18 h	24 h	24 h
	Microvascular	20 h	18 h	7days	7 days

Acceleration of the pseudovessel rearrangement induced by infection with *B. henselae* strains or their supernatants was observed only with human microvasculature-derived ECs (HSkMEC). In feline cells, bacterial infection accelerates the network destruction. Noticeably, uninfected human ECs from micro- and macro-vasculature display differences in the angiogenesis kinetics.

Similar results were obtained when ECs were treated by corresponding *B. henselae* supernatants.

Results from one out of four independent experiments are shown.

Human Macrovascular EC: immortalized Human Umbilical Vein Endothelial Cells (iHUVEC); Human Microvascular EC: Human Skin Microvascular Endothelial Cells (HSkMEC); Feline Macrovascular EC: Feline Umbilical Vein Endothelial Cells (FOmEC); Feline Microvascular EC: Feline Skin Microvascular Endothelial Cells (FSkMEC).

**Figure 2:**
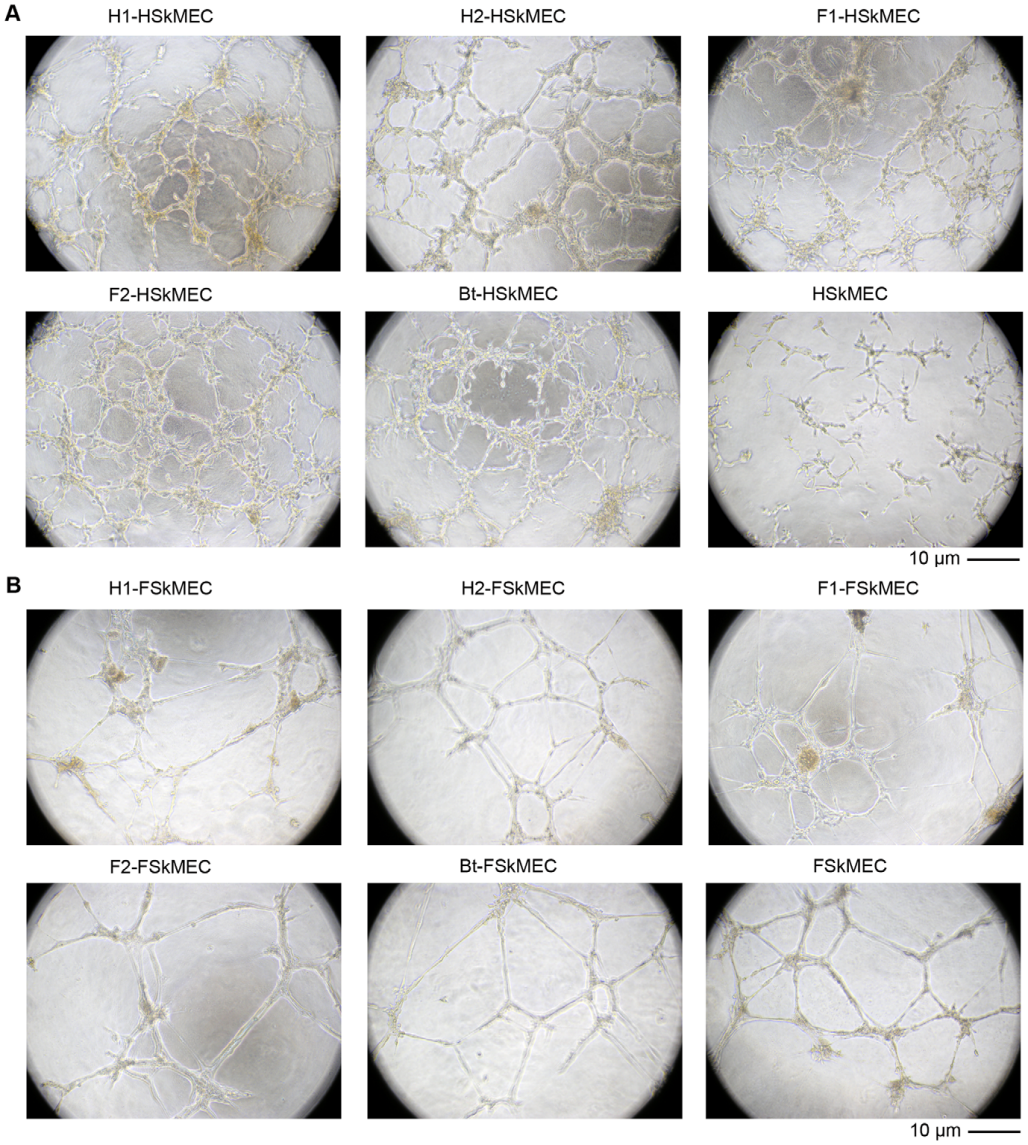
Effect of various *Bartonella* strains on human and feline ECs capillary-like structure formation on Matrigel™. HSkMEC (Human Skin Microvascular Endothelial Cells) infection by *B. henselae* (multiplicity of infection: 100 bacteria/cell) increases capillary-like structure formation as compared to uninfected cells (A). In contrast, *B. henselae* infection has no visible effect on FSkMEC (Feline Skin Microvascular Endothelial Cells) (B). Pictures have been taken 10 h and 20 h after seeding of FSkMEC and HSkMEC, respectively. Original magnification: 50x. H1: *B. henselae* human type I strain (reference strain Houston-1 ATCC 49882); H2: *B.henselae* human type II strain (Marseille); F1: *B. henselae* feline type I strain (F1 297172); F2: *B. henselae* feline type II strain; Bt: *B. tribocorum*.

In parallel, we monitored the effects of *Bartonella* (*B. henselae* genotype I/II and *B. tribocorum*) infection on the four cell lines. Irrespective of the species of origin and genotypes of *Bartonella,* infected HSkMEC efficiently achieved angiogenesis in terms of tube formation, extended projections morphology, branching points and number of cells as compared to uninfected cells (Fig. 2 A). The influence of *Bartonella* infection on the formation of the capillary-like network by HSkMEC was dose-dependent (data not shown). Infection accelerated angiogenesis by few hours in Human Skin Microvascular cells (table 1).

**Figure 3:**
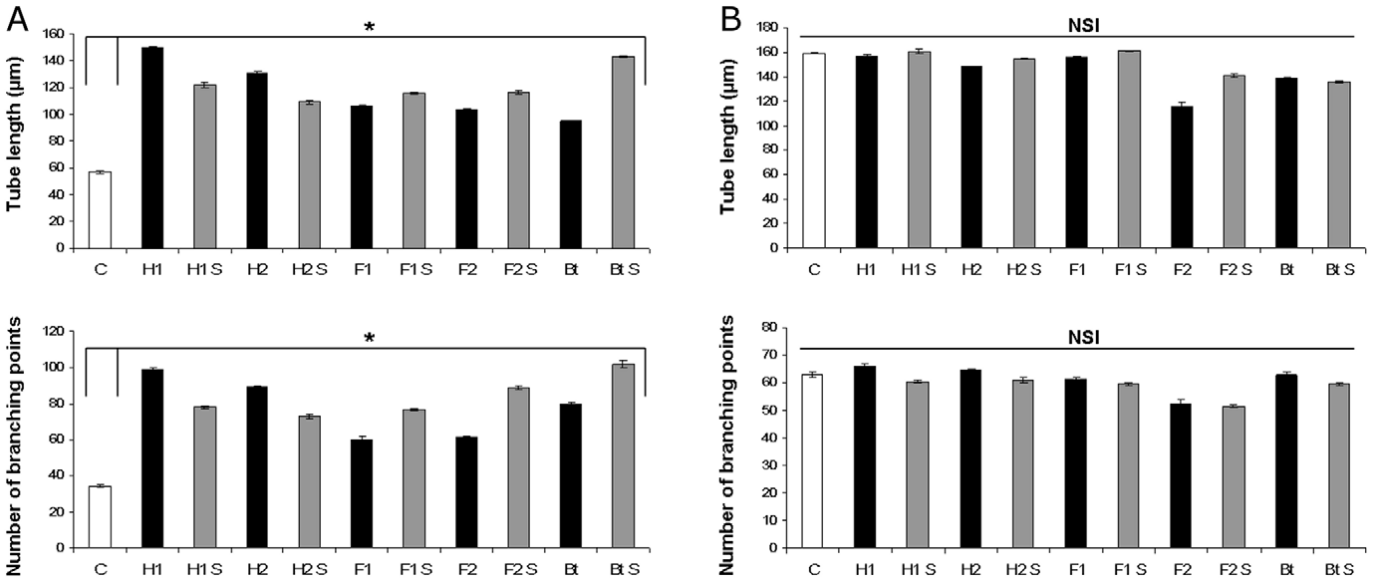
Quantitative analysis of capillary-like structure formation by cell projection length and branching point number determination. Human Skin Microvascular ECs (HSkMEC) infection by *B. henselae* strains (M.O.I.of 100, black bars) or treatment by their culture supernatants (250 µg/ml, grey bars) increases angiogenesis assessed by cell projection length and branching point number when compared to uninfected cells (white bars) (measures performed 20 hours p.i.) (A). In contrast, Feline Skin Microvascular ECs (FSkMEC) infection by *B. henselae* or treatment by their culture supernatants has no such effect (measures performed 10 hours p.i.) (B). Data from four independent experiments are shown as mean ± standard deviation (*p<0.05 *versus* uninfected or untreated controls). NSI: No Significant Increase. C: uninfected or untreated controls cell; S: *Bartonella* culture supernatant; H1: *B. henselae* human type I strain (reference strain Houston-1 ATCC 49882); H2: *B. henselae* human type II strain (Marseille); F1: *B. henselae* feline type I strain (F1 297172); F2: *B. henselae* feline type II strain; Bt: *B. tribocorum*.

Importantly, kinetics of angiogenesis of the FSkMEC was not changed upon *Bartonella* infection whatever strain used (Fig. 2B, Fig. 3B and table 1). Angiogenesis of FSkMEC and FOmEC started 2 h after seeding on Matrigel™. The destruction of the network was slightly faster in the infected feline cell lines (18 hours) as compared to uninfected cells (20 hours) (table 1). No effect was observed upon infection on the structure of the newly formed vessels (Fig. 2B and Fig. 3B). Importantly, as a negative control, no pro-angiogenic effect was observed, both on human and feline ECs with *E. coli* or with heat-inactivated *B. henselae* (data not shown).

The observed effects were quantified. As shown on Fig. 3A, *Bartonella*-infection increased capillary-like formation by HSkMEC by a factor 1.8 to 2.6 as compared to uninfected ECs (*p*<0.05) and the number of branching points by a factor of 1.78 to 3 (*p*<0.05) (Fig. 3 A). This network was observed until 7 days with these cells, and then disappeared (table 1).

Capillary-like formation was increased in iHUVEC by a 2 fold upon infection such as in HSkMEC. However, as opposed to HSkMEC, *B. henselae* infection did not speed this effect (table 1). The network was observed only until 24 hours, before resorbing (table 1).

Thus, the effect of *Bartonella-*induced angiogenesis was more potent in human ECs than feline ECs (where infection even accelerated network destruction). Moreover, capillary-like structures were markedly more durable in micro- *versus* macro-vasculature ECs.

### Angiogenic response of ECs to *Bartonella* culture supernatants

In order to investigate the effects of *Bartonella* secretome, different *Bartonella* culture supernatants were generated and tested for their ability to induce angiogenesis in human and feline ECs. *Bartonella* culture supernatants did induce tube formation in human ECs. The pro-angiogenic responses obtained with *Bartonella* culture supernatants were accelerated by a factor 1.9 to 2.5 (*p*<0.05), as in *Bartonella*-infected human ECs (Fig. 3A). The resulting effect of *Bartonella* or their culture supernatants on the morphology of human ECs was similar, *i.e.* ECs appeared elongated with long membrane projections (data not shown).

In the system of feline endothelial cells, *Bartonella* culture supernatants, as bacteria themselves, did not influence angiogenesis (Fig. 2B and Fig. 3B).

### Wound-induced migration of human ECs by *Bartonella* infection

**Figure 4:**
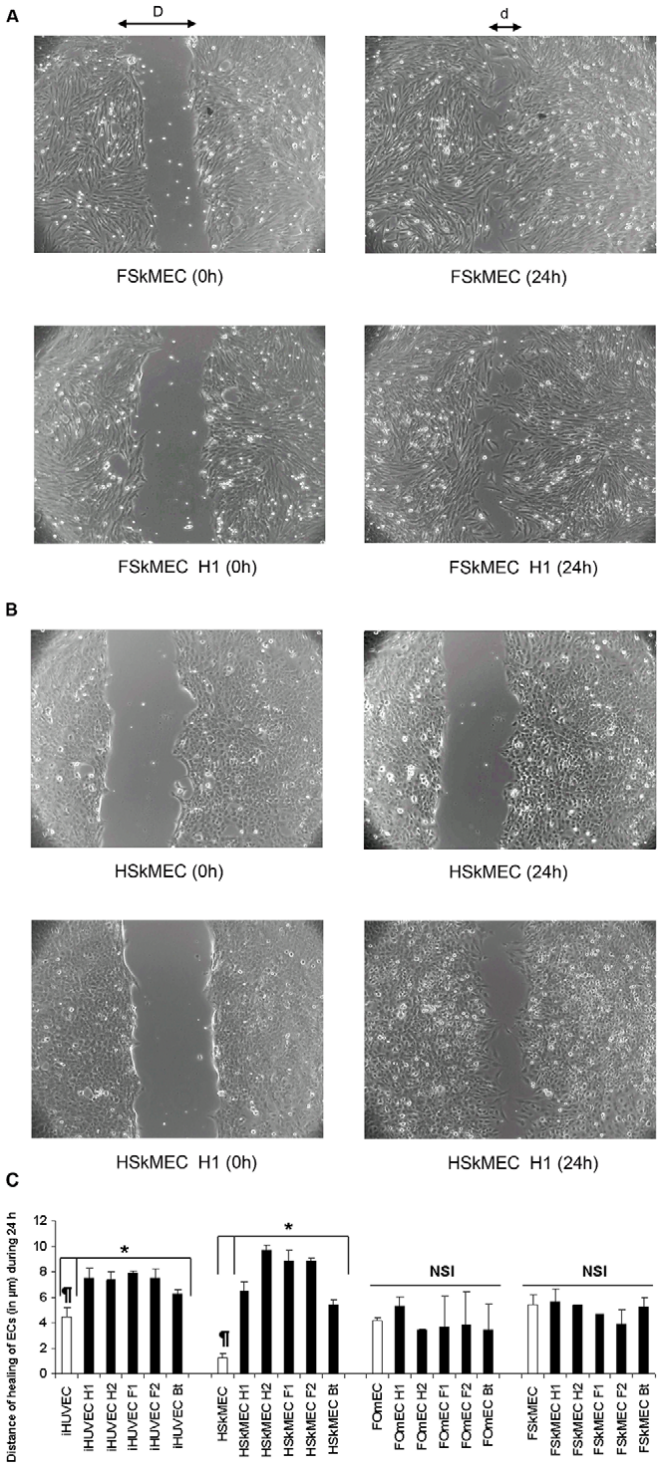
Effect of human and feline ECs *Bartonella* infection on cell migration assessed in wound healing assay. A: Healing test (FSkMEC and FSkMEC-H1) Healing distance  =  D − d. B: Healing test (HSkMEC and HSkMEC-H1) C: Quantification of the effect of *Bartonella* infection on wound healing *B. henselae* infection increased strongly human Skin Microvascular ECs (HSkMEC) migration, in a less extent human macrovascular ECs (iHUVEC) migration but did not affect feline ECs (FOmEC and FSkMEC). Results show mean ± standard deviation from three independent experiments. (iHUVEC *versus* HSkMEC controls: ¶p = .015; infected cells *versus* uninfected cells: *p<0.05). NSI: No Significant Increase. H1: *B. henselae* human type I strain (reference strain Houston-1 ATCC 49882); H2: *B. henselae* human type II strain (Marseille); F1: *B. henselae* feline type I strain (F1 297172); F2: *B. henselae* feline type II strain; Bt: *B. tribocorum*.

As ECs migration is an essential step for angiogenesis, the significance of the site of infection in term of endothelium susceptibility was examined. The endothelial cell reaction according to their tissue origin was followed upon *Bartonella* infection by measuring their migration speed to heal a mechanical wound (Fig. 4A and 4B). In uninfected cells (at 24 h), wound-healing was at least twice faster in iHUVEC than in HSkMEC (4.5±0.7 and 1.25±0.35 µm within 24 h, respectively with *p* = 0.015) (Fig. 4C). Infection speeded the wound-healing for both iHUVEC and HSkMEC. Remarkably, in iHUVEC, healing speed was approximately doubled, whereas in HSkMEC, it was increased by a factor of 4.3 up to 7.8 (*p*<0.05) (Fig. 4).

In feline ECs (FOmEC and FSkMEC) wound-induced migration was recorded and no stimulation by *Bartonella* infection was observed (Fig. 4A and Fig. 4C).

### Effect of *Bartonella* infection on human and feline ECs on cAMP production

**Figure 5:**
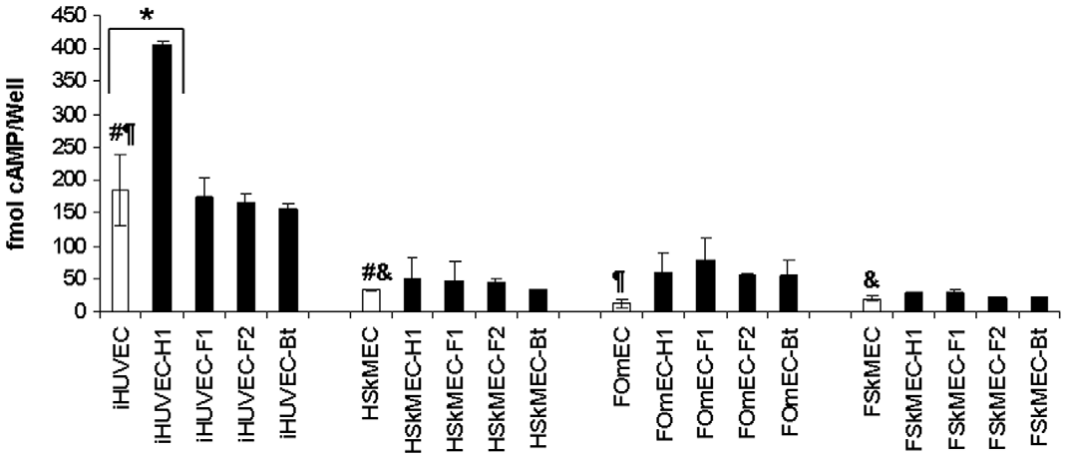
Effect of *B. henselae* infection on intracellular cAMP production in human and feline EC lines. *B. henselae* infection did not significantly increase cAMP production by ECs, except for iHUVEC infected by H1 strain. Results were monitored 30 h post infection with bacteria (M.O.I. of 150) and expressed in fmol/well as mean ± standard deviation (*p<0.05 for the differences between infected cells *versus* uninfected cells; #, ¶ and & p<0.05, for the differences between uninfected cells). H1: *B. henselae* human type I strain (reference strain Houston-1 ATCC 49882); F1: *B. henselae* feline type I strain (F1 297172); F2: *B. henselae* feline type II strain; Bt: *B. tribocorum*. iHUVEC: immortalized Human Umbilical Vein Endothelial Cells; HSkMEC: Human Skin Microvascular Endothelial Cells; FOmEC: Feline Umbilical Vein Endothelial Cells; FSkMEC: Feline Skin Microvascular Endothelial Cells.

It was shown in the HUVEC model that *B. henselae* genotype I (reference strain Houston-1) triggered the production of cAMP [29]. As, according to these authors, anti apoptosis was mediated through increased cAMP levels, this induction was assessed in ECs upon infection by distinct *Bartonella* strains. In uninfected ECs of the macro- or the micro-vasculature, the levels of cAMP produced by the feline ECs (FOmEC [12.06±7.13 fmol] and FSkMEC [19.38±4.11 fmol]) were significantly weaker than in human ECs (iHUVEC [203.57±57.66 fmol] and HSkMEC [32.68±1.80 fmol]) (*p*<0.05) (Fig. 5). Moreover, cAMP production by human ECs of the macrovasculature (iHUVEC) was higher than microvasculature-derived ECs (HSkMEC) by a factor of 6.2 (203.57±57.66 *vs* 32.68±1.80 fmol respectively with *p* = 0.0004). In feline ECs, the low levels of production did not allow to detect any significant difference between ECs of the macro- and the micro-vasculatures (Fig. 5).

Upon infection, cAMP increased only in human macrovasculature ECs (iHUVEC) infected by strain H1 by a factor of 2.2 (reaching 405.16±6.72 fmol) (*p*<0.05). Strains F1, F2 and Bt did not induce any change. Human Skin Microvasculature ECs were not affected by any of the strains.

Feline ECs from micro- or macro-vasculature ECs did not react to the bacterial infection (Fig. 5).

### *Bartonella* increases VEGF production in human microvascular ECs

The involvement of VEGF in *B. henselae* effect on angiogenesis was investigated. We took into account: a) the origin of the bacterial strains (human *versus* feline); b) the bacterial genotype (I *versus* II); c) the ECs species (human *versus* feline), and d) their type (macro *versus* micro vessels derived-ECs).

**Figure 6:**
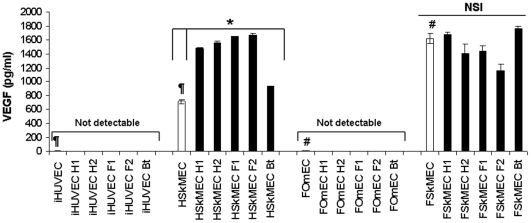
VEGF production in human and feline macro- and micro-vascular ECs upon infection with *Bartonella* strains. *B. henselae* triggers VEGF production in Human Skin Microvascular ECs (HSkMEC) but not in human macrovascular ECs (iHUVEC) or in feline macro- (FOmEC: Feline Umbilical vein Endothelial Cells) or micro- (FSkMEC: Feline Skin Microvascular Endothelial Cells) vascular ECs. In addition, a clear differential production of VEGF between micro- and macro-vascular ECs is shown both in human and in cat. VEGF level was determined by ELISA after 72 h of infection and data shown as mean ± standard deviation (*p<0.05 infected *versus* uninfected cells; ¶ and #p<0.0015 for the differences between uninfected cells). NSI: No Significant Increase. iHUVEC: immortalized Human Umbilical Vein Endothelial Cells; HSkMEC: Human Skin Microvascular Endothelial Cells; FOmEC: Feline Umbilical Vein Endothelial Cells; FSkMEC: Feline Skin Microvascular Endothelial Cells. H1: *B. henselae* human type I strain (reference strain Houston-1 ATCC 49882); H2: *B. henselae* human type II strain (Marseille); F1: *B. henselae* feline type I strain (F1 297172); F2: *B. henselae* feline type II strain; Bt: *B. tribocorum*.

A differential production of VEGF was measured between non infected microvascular and macrovascular ECs both of human and feline origin as shown on Fig. 6. Human and feline microvascular ECs produced high levels of VEGF [717.88±32.50 and 1623.19±72.81 pg/ml respectively] as compared to human and feline macrovascular ECs [3.75±3.12 and 6.88±5.0 pg/ml respectively, p = 0.001] (Fig. 6).

An increase in VEGF production by *B. henselae* infection was induced only in human microvascular skin-derived cells (HSkMEC) (Fig. 6).

Interestingly, the four *B. henselae* strains significantly increased VEGF production by HSkMEC by a factor of 2.1 to 2.4 while *B. tribocorum* was less effective (factor of 1.3) (*p*<0.05). Feline microvascular cells production of VEGF was not significantly affected by the various strains.

### Effects of *B. henselae* infection on VEGFR-2 activation

**Figure 7:**
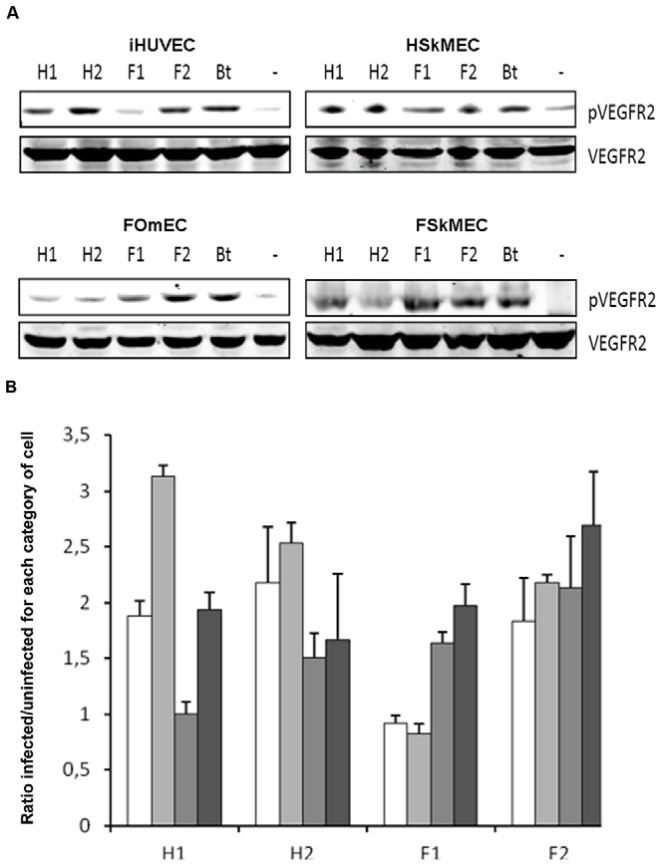
*B. henselae* -mediated VEGFR-2 activation on ECs. **A:** Homologous type of *B. henselae* -mediated VEGFR-2 activation on ECs. *B. henselae* strains from human origin (H1 and H2) mostly stimulated VEGFR-2 phosphorylation in human ECs (HUVEC an HSkMEC) and the strains from feline origin (F1 and F2) stimulated VEGFR-2 phosphorylation in feline ECs (FOmEC and FSkMEC). Western blot analysis of VEGFR-2 and phosphorylated VEGFR-2 (pVEGFR-2) following a 24 hours infection of ECs by *B. henselae* were shown as one representative experiment out of three. iHUVEC: immortalized Human Umbilical Vein Endothelial Cells; HSkMEC: Human Skin Microvascular Endothelial Cells; FOmEC: Feline Umbilical Vein Endothelial Cells; FSkMEC: Feline Skin Microvascular Endothelial Cells. H1: *B. henselae* human type I strain (reference strain Houston-1 ATCC 1 49882); H2: *B. henselae* human type II strain (Marseille); F1: *B. henselae* feline type I strain (F1 297172); F2: *B. henselae* feline type II strain; Bt: *B. tribocorum*; - : Uninfected cells. **B:** Quantitative analysis of *Bartonella*-mediated VEGFR-2 activation on ECs. *Bartonella* strains effect on VEGFR-2 activation expressed as levels increase over uninfected controls. ECs (iHUVEC, HSkMEC, FOmEC and FSkMEC) infection by *Bartonella* was associated with an increased phosphorylation of VEGFR-2. Serie 1 (White bar): iHUVEC: immortalized Human Umbilical Vein Endothelial Cells. Serie 2 (Light grey bar): HSkMEC: Human Skin Microvascular Endothelial Cells. Serie 3 (Dark grey bar): FOmEC: Feline Umbilical Vein Endothelial Cells. Serie 4 (Black bar): FSkMEC: Feline Skin Microvascular Endothelial Cells. H1: *B. henselae* human type I strain (reference strain Houston-1 ATCC 49882); H2: *B. henselae* human type II strain (Marseille); F1: *B. henselae* feline type I strain (F1 297172); F2: *B. henselae* feline type II strain.

The level of activation of the VEGF receptor VEGFR-2 was estimated by monitoring VEGFR-2 phosphorylation in Western-blot. Figure 7 indicates that ECs (iHUVEC, HSkMEC, FOmEC and FSkMEC) infection by *Bartonella* was associated with an increased phosphorylation of VEGFR-2.

In addition, the level of the VEGFR-2 phosphorylated form was significantly higher by a 2 fold when ECs were infected by a strain originating from a homologous host (*i.e.* when human ECs were infected by *B. henselae* strains originating from humans and when feline ECs were infected by *B. henselae* strains originating from cats). In particular, VEGFR-2 phosphorylation induced in human ECs (HSkMEC and iHUVEC) by strain H1 was respectively 2.2 to 3.3 higher than that induced by F1. Conversely, VEGFR-2 phosphorylation induced in Feline ECs (FSkMEC and FOmEC) by H1 was lower than that induced by F1 respectively 0.95 to 0.6 (Fig. 7A and 7B). *B. tribocorum* induced a comparable activation of VEGFR-2, whatever the origin of cell lines (Fig. 7A).

## Discussion

*B. henselae* is a facultative intracellular pathogen associated with the induction of vasoproliferative tumors in humans (bacillary angiomatosis and peliosis) that are not observed in cats (host reservoir) [4], [6], [7], [8].

Our original cellular models allowed us to reveal intrinsic differences in the angiogenesis kinetics and the wound-healing process between human micro- and macro-vasculature-derived ECs. Importantly, our results strongly suggest that effects of *B. henselae* on microvasculature-derived cells strongly differ from macrovasculature ones.

In human cells, *Bartonella* infection affected the angiogenic process at two different levels: a) a slight acceleration of the pseudovessel rearrangement was observed, only with microvasculature-derived ECs (Table 1) and b) the number of pseudo-vessels was increased in both micro- and macro-vasculature-derived ECs (figure 3).

One key point of our findings concerns feline ECs from both micro- and macro-vasculatures. Indeed, not only *B. henselae* had no visible pro-angiogenic effect *in vitro*, but the infection seemed to accelerate the network destruction of the feline cells. These results might recapitulate the clinical situation, *i.e.* the absence of bacillary angiomatosis in cats (even in FIV and/or FeLV infected cats) *versus* bacillary angiomatosis in humans and even in dogs as recently described [40].

Hence, our cellular models are relevant to understand the cellular and molecular bases of the pro-angiogenic potential of *B. henselae* and to explore the mechanisms underlying bacillary angiomatosis/peliosis.

This model of infection shows that, independently of the bacterial species (*B. henselae vs B. tribocorum*), or of the *B. henselae* genotype (I or II), *Bartonella* tested here can induce *in vitro* angiogenesis. Interestingly, *B. tribocorum* has no known pro-angiogenic potential *in vivo*. Moreover we did not detect any obvious difference of activity between the different strains of *Bartonella* tested contrary to the work of Chang et *al* [30].

Furthermore, the stimulatory effect of *B. henselae* is not dependent upon attachment or penetration in the target cell suggesting that *B. henselae* may produce and secrete endothelial cell-stimulatory factor(s).

In addition, the inactivation of *B. henselae* by heat treatment abolished its vasoproliferative activity, suggesting that the angiogenic effect of *B. henselae* on ECs require live bacteria.

Previous studies dedicated to understand the interactions between primary HUVEC and *B. henselae* Houston 1 (genotype 1) have suggested a correlation between the anti-apoptotic activity of *B. henselae* and an increased cAMP production [29]. Remarkably, in our study, the stimulation of cAMP production was observed only when *B. henselae* Houston 1 was inoculated to iHUVEC. Such effect was never obtained when skin-derived EC - HSkMEC –, which are directly involved in bacillary angiomatosis, were used, even when they were infected by Houston 1. This points to the fact that macrovasculature-derived ECs (iHUVEC/HUVEC) deeply differ from microvasculature ECs [31], [32], [33], [41], [42]. Remarkably, in our study, cAMP production does not reflect ECs proliferation in the case of infected microvascular ECs. According to these results, microvascular skin endothelial cells- HSkMEC, clearly represent a better model for clinical bacillary angiomatosis.

Feline macro- and/or micro-vasculature ECs cAMP production was weak and not related to infection.

Such low level of cAMP production by human microvasculature ECs (HSkMEC) does not corroborate the pro-angiogenic effect of *B. hensela*e interaction with human ECs. Nishihara et *al* [43], have shown that the effects of cAMP are highly cell type-specific. cAMP either promotes or antagonizes apoptosis in a cell type, environment- and stimulus- related manner. These, together with our results, illustrate that the model based on HUVEC which is not the clinical cell target, does not allow any definitive conclusion about the role of cAMP during *B. henselae*- anti-apoptotic activity.

VEGF, one of the main pro-angiogenic factors, plays a critical role in blood vessel formation [44] and pathological angiogenesis [45]. This factor seems also to be involved in *Bartonella*-induced angiogenesis. For instance, in *verruga peruana* (induced by *B. baciliformis*), the primary source of vascular endothelial growth factor (VEGF) is the epidermis [46] and endothelium of verruga peruana expresses VEGF receptors (VEGFR1 and VEGFR2) [46].

In addition, while HUVEC did not produce VEGF in response to *B. henselae,* infection promotes their proliferation [22]. Further study by Kempf et *al* showed the importance of VEGF in *B. henselae*-mediated ECs proliferation although it appeared not to be produced by ECs only. Overall, this strongly suggests that a paracrine loop could be involved in *B*. *henselae* VEGF action [27], [28]. However, up to now, the majority of these studies have been carried out on macrovascular ECs (i.e. HUVEC). Interestingly, using our species- and organ-specific ECs model, we showed that *B. henselae* increased VEGF production by Human Skin Microvascular ECs but not by iHUVEC or feline macro- or micro-vascular ECs. These results, which might explain the decreased pathogenic potential of *B. henselae* infection for cat as compared to human, strongly suggests the involvement of an autocrine secretion of VEGF by skin ECs. This finding points out that the mechanism of *B. henselae*-mediated angiogenesis induction implies autocrine VEGF production and stimulation of the infected endothelium.

In parallel to VEGF production, the phosphorylation of VEGFR-2 was observed in ECs upon infection, mostly by homologous strains. This phenomenon is of high interest in the context of epidemiological human infection. As *B. henselae* isolates infecting a human have always a feline origin, it is tempting to speculate that an adaptive switch is taking place upon cat scratch in humans. Altogether, our results raise the hypothesis of host-dependent signaling in ECs upon bacterial infection as VEGFR-2 activation is more prominent in human ECs exposed to homologous strains. The same holds true for feline ECs. Because bacillary angiomatosis is not detected in cats infected by homologous strains, alternate mechanisms are likely taking place, Our results support the fact that VEGF signaling might be increased in human ECs through an autocrine production, which does not occur in feline ECs. Paracrine VEGF activation cannot be excluded; therefore, it will be interesting to further investigate whether VEGF can be released in the perivascular microenvironment by other cells, such as macrophages and polymorphonuclear cells. Our results on VEGF and VEGFR-2 are in apparent contradiction with those obtained by Scheidegger et *al* [47]. However, the concentration ranges and source of VEGF were far for being comparable.

Our study highlights the ECs organo- and species- specificity in their interactions with *B. henselae.* Indeed, our model further demonstrates the difference of reactivity displayed by human *vs* feline ECs on the one hand and by the macro- *vs* the micro-vasculature on the other hand. Additionally, local physiological microenvironment is most likely to play an important role. In this context, our cellular systems will allow studying the process of specific ECs infection by *B. henselae*. They will offer new possibilities to investigate bacterial and cellular factors which determine human *vs* cat reactivity, as well as the mechanisms involved in anti-apoptotic and/or pro-angiogenic effects, ultimately resulting to bacillary angiomatosis and peliosis only in humans.

In conclusion, according to the validated hypothesis stating the organospecificity of the endothelium [31], [36], [37], [38], our work extends this concept to the species-specific endothelial cell phenotypes. In this scenario, ECs could act as a major reservoir for infectious pathogens. Moreover, at a second level the ECs from the same tissue but from different species display similar organospecificity but distinct responses to infection. This is clearly illustrated by the reactivity of the human skin-derived ECs compared to the feline skin-derived ECs. It will further be necessary to evidence the molecular mechanism by which feline ECs are recognized but not activated upon binding of *B henselae,* while human ECs are activated to grow and proceed to tumor-like state. The co-activation signaling should indicate differential gene induction and regulation processes.

Such information will provide new insights into the mechanisms of species-specific tolerance *versus* pathologic infections as observed in AIDS malignancies.
